# Magnitude of rise in proneurotensin is related to amount of triglyceride appearance in blood after standardized oral intake of both saturated and unsaturated fat

**DOI:** 10.1186/s12944-020-01361-0

**Published:** 2020-08-21

**Authors:** Ayesha Fawad, Celine Fernandez, Andreas Bergmann, Joachim Struck, Peter M. Nilsson, Louise Bennet, Marju Orho-Melander, Olle Melander

**Affiliations:** 1 Department of Clinical Sciences, Skåne University Hospital, Lund University, Malmoe, CRC, Jan Waldenstroems gata 35, bldg 91, level 12, 214 28 Malmoe, SE Sweden; 2 Sphingotec GmbH, Hennigsdorf, Germany and the Waltraut Bergmann Foundation, Hohen Neuendorf, Germany; 3 Departement of Emergency and Internal Medicine, Skåne University Hospital, Malmö, Sweden

**Keywords:** Obesity, Fat absorption, Gut hormones, Triglycerides, Proneurotensin, Type 2 diabetes

## Abstract

**Background:**

In rodents, neurotensin contributes to high fat diet induced obesity by facilitation of intestinal fat absorption. The effect of oral lipid load on plasma proneurotensin and relationship with plasma triglycerides in humans is unknown.

**Aim:**

To investigate the acute effects of an oral lipid load on proneurotensin and plasma triglycerides and their interrelationships in healthy individuals.

**Setting/ methods:**

Twenty-two healthy subjects were given 150 mL of full milk cream (54 g fat) and 59 mL of pure olive oil (54 g fat) in the fasted state at two different occasions separated by at least 1 week in random order. Venous blood was drawn at fasted before 0 h (h) and at 1 h, 2 h and 4 h after ingestion. Post-ingested values of proneurotensin and plasma triglycerides were compared with fasting levels and post ingestion Area Under the Curve (AUC) of proneurotensin was correlated with that of plasma triglycerides.

**Results:**

An immediate rise of plasma proneurotensin and plasma triglycerides were observed after ingestion of cream with maximum increase at 2 h for proneurotensin [mean (95% confidence interval)] of 22 (12–31) pmol/L *(P < 0.001)* and at 3 h for triglycerides of 0.60 (0.43–0.78) mmol/L *(P < 0.001).* Similarly, plasma proneurotensin and plasma triglycerides increased after ingestion of olive oil with maximum increase of proneurotensin at 3 h of 62 (46–78) pmol/L *(P < 0.001)* and plasma triglycerides at 3 h of 0.32 (0.18–0.45) mmol/L *(P < 0.001).* The post lipid load AUC for proneurotensin correlated significantly with the AUC for plasma triglycerides both after cream (*r* = 0.49, *P = 0.021*) and olive oil (*r* = 0.55, *P = 0.008*), respectively.

**Conclusion:**

Proneurotensin increases after an oral lipid load of both cream and olive oil and the rise of post-ingestion plasma triglycerides is significantly related to the rise of post-ingestion proneurotensin.

## Background

The global increase in the incidence and prevalence of obesity is occurring in parallel with the burden of cardiovascular disease (CVD) and metabolic diseases such as type 2 diabetes, hypertension and dyslipidemia. Large prospective and observational studies have already confirmed the adverse effects of obesity and dyslipidemia on CVD [1]. However, prevention of significant numbers of CVD and residual risk factors remains unsettled. As obesity is an important modifiable risk factor for both diabetes and for diabetes-related CVD, novel mechanisms underlying obesity, might thus contribute to new ways for prevention of cardiometabolic diseases [2, 3].

Levels of plasma triglycerides, both in the fasted and the non-fasted state, are higher in diabetes patients with insulin resistance than in diabetes patients without insulin resistance [4], and this has been suggested to be an independent CVD risk factor [4, 5].

Neurotensin (NT) is a 13-amino acid peptide expressed primarily in the brain and neuroendocrine cells of the small intestine. It is assumed that the absolute majority of the NT that is measurable in blood comes from the periphery, i.e. from the small intestine, where it is stimulated by dietary fat intake [6].

In the brain, NT acts as a neurotransmitter and regulates different functions like temperature regulation, nociception, pituitary hormone secretion and dopaminergic transmission [7]. In the small intestine, neuroendocrine cells secretes NT in response to fat ingestion, and in animal studies, neurotensin has shown to be instrumental to promote fat absorption from the small intestine into the blood stream [8, 9].

Research in recent years have shown that elevated fasting level of proneurotensin, i.e. a peptide derived from the NT precursor hormone that is release in parallel with the mature peptide [10], predicts future development of obesity, diabetes mellitus, CVD and premature mortality. Moreover, high level of proneurotensin among obese subjects is strongly linked to fat accumulation in the liver and related to both the presence and severity of Non-Alcoholic Fatty Liver Disease (NAFLD) [11]. Interestingly, rodent models who were genetically deficient of NT, absorbed less fat from the small intestine and were protected from diet induced obesity, insulin resistance and hepatic steatosis as compared to animals with intact NT production [9]. In line with this finding, an identical phenotype was observed in rodents lacking one of the key receptors of NT, i.e. NT-receptor 3 (NTSR3) [12]. Thus, in animals, NT seems to promote intestinal fat absorption, high fat diet induced obesity and liver steatosis. In humans, circulating NT rapidly rises several minutes after a meal enriched in fatty acid [10]. It was demonstrated that NT acts as a hormone released from the small intestine following fat ingestion and that it facilitates fat digestion by stimulating pancreatic secretions in rats [13]. Collectively these data suggest that NT (in humans commonly proxy-measured by the stable proneurotensin peptide) promotes intestinal uptake of fat and promotes its central storage in the liver which in turn contributes to obesity, NAFLD, diabetes and CVD at high levels of fat intake. Importantly, although it is well known that NT rises after fat intake in both animals and human but no human study has examined whether post-lipid ingestion rise in NT actually contributes to intestinal uptake of lipids. In order to study a plausible mechanism linking high NT to obesity and its sequels; it is tested if the rise of post-ingestion plasma triglycerides after cream and olive oil is significantly related to the rise of post-ingestion proneurotensin.

## Methods

In this study, 22 healthy young subjects (10 men and 12 women) without any previous medications and diseases volunteered to participate. Trained research nurses conducted the standard physical examinations. It involved the measurement of blood pressure, weight, height, waist circumference, as well as collection of blood samples. Blood pressure was measured in the supine position after 5 min rest with the arm at the level of heart. The mean of the two measurements, taken 1 min apart, was calculated. Height was measured to the nearest cm. Weight is measured to the nearest kg in subjects wearing light clothes, without shoes. BMI (kg/m^2^) was calculated as weight (kg) divided by height (m) squared. Subjects were classified according to BMI using WHO classification: normal weight: BMI < 25 kg/m^2^; overweight: BMI ≥ 25 kg/m^2^ and < 30 kg/m^2^; and obese: BMI ≥ 30 kg/m^2^.

Plasma proneurotensin was measured from stored fasting plasma, frozen at − 80 °C and stored immediately after sampling. Proneurotensin assays were performed blinded to clinical data at an independent laboratory (ICI immunochemical Intelligence GmbH, Berlin, Germany) by using a one-step sandwich immunoassay based on a chemiluminescence label and coated-tube technique (SphingoTec©, Hennigsdorf, Germany). The limit of detection of proneurotensin precursor fragment was 1.9 pmol/L [14, 15]. Cholesterol and triglycerides in serum were analyzed using enzymatic methods (Bayer Diagnostics, Tarrytown NY, USA) [16]. HDL-cholesterol in serum was measured enzymatically after isolation of LDL and VLDL (Boehringer Mannhein GmbH, Mannheim, Germany) and LDL-cholesterol was estimated using Friedewald’s method [17]. Serum insulin levels were determined using the Radioimmunoassay technique (Access© Ultrasensitive Insulin, Beckman Coulter, Bellport, NY, USA) [18].

The inter assay CV for proneurotensin was 6.2% at 48 pmol/L and 4.1% at 191 pmol/L.

Participants received milk cream and olive oil at two separate occasion and all routine plasma laboratory analyses were performed at the University Hospital’s central clinical laboratory by using certified methods.

After over-night fast (since 22:00 evening before the test day), all 22 subjects underwent ingestion of 150 mL of milk cream (54 g fat) and 59 mL of pure olive oil (54 g fat) at two different occasions in random order, with the two oral lipid loads separated by 1 week. Blood samples were taken for measurement of plasma triglycerides, proneurotensin and blood glucose in the fasted state and after every hour for 4 h.

### Statistical analysis

Clinical characteristics at the first visit were presented as means ± standard deviations (SD) for continuous variables and as numbers and percentage for dichotomous variables. The individual hourly plasma concentration values of proneurotensin, triglycerides and glucose after the respective oral lipid load was compared to the fasted value (post lipid load value minus fasted value) using paired t-test and the delta values were presented as means and 95% confidence intervals. The change at the maximal post-lipid ingestion concentration of proneurotensin and triglycerides versus the baseline concentration was calculated. Post lipid ingestion areas under the Curve (AUC) of plasma proneurotensin and triglycerides were calculated and correlated the AUCs of proneurotensin and triglycerides using Pearson correlation analysis. Differences were considered statistically significant when two-tailed *p*-values were less than 0.05.

## Results

**Fig. 1:**
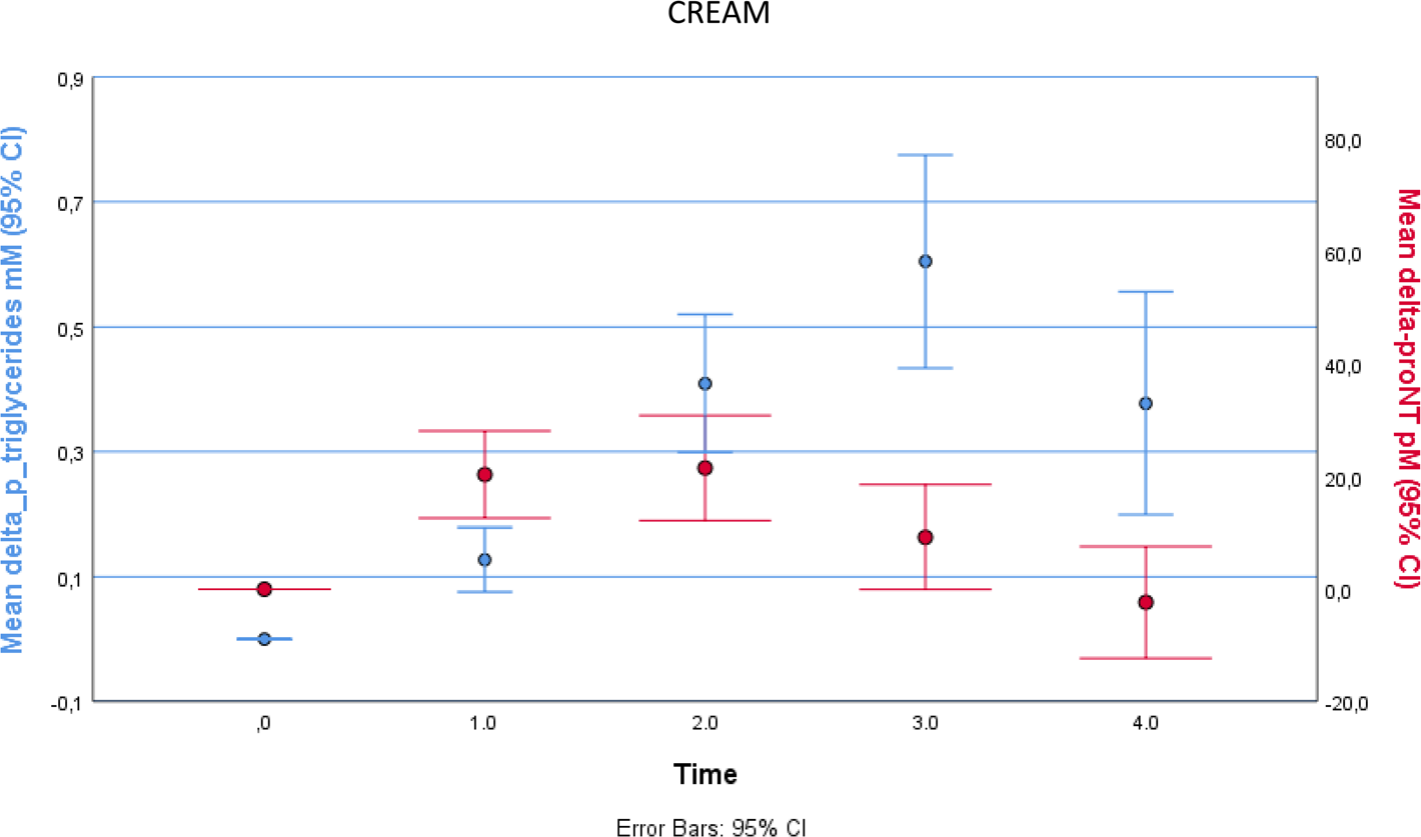
Changes of proneurotensin and triglycerides after ingestion of cream and correlation of post lipid load Area Under the Curves (AUC) for proneurotensin with the AUC for plasma triglycerides after cream

The baseline (first visit) clinical characteristics of the study subjects are shown in Table 1, indicating healthy status. Dynamic changes of proneurotensin and triglycerides after ingestion of cream and olive oil, respectively, are shown in Fig. 1. After ingestion of cream, proneurotensin in plasma rose significantly after 1 h and reached its maximum at 2 h [mean (95% confidence interval)] of 22 (12–31) pmol/L *(P < 0.001)* after which it declined. Triglycerides also rose significantly after 1 h and reached its maximum after 3 h [0.60 (0.43–0.78)] mmol/L (*P* < 0.001) before declining. Similarly, after ingestion of olive oil, proneurotensin rose significantly after 1 h and reached its maximum values after 3 h [62 (46–78) pmol/L] *(P < 0.001)*. Triglycerides rose significantly after 2 h and reached its maximum after 3 h 0.32 (0.18–0.45) mmol/L] *(P < 0.001).* After cream, the AUC for proneurotensin and triglycerides are 49 ± 74 pmol/L x h and 1.5 ± 0.95 mmol/L x h respectively. After olive oil, the AUC for proneurotensin and triglycerides are 196 ± 115 pmol/L x h and 0.71 ± 0.77 mmol/L x h respectively. The AUC of both proneurotensin and triglycerides differed significantly between cream and olive oil (Table 2).

**Table 1:** Anthropometric and blood parameters of study participants

Parameter	Mean ± SD
N (men/women)	22 (10/12)
Age (years)	31 ± 9.5
Proneurotensin (pmol/L)	122 ± 48
Systolic bloodpressure (mmHg)	115 ± 16
Diastolic bloodpressure (mmHg)	66 ± 9
Cholesterol (mmol/L)	4.7 ± 0.97
HDL Cholesterol (mmol/L)	1.7 ± 0.28
LDL Cholesterol (mmol/L)	2.9 ± 0.82
Insulin (mIE/L)	7.1 ± 4.0
Glucose (mmol/L)	5.2 ± 0.53
Triglycerides (mmol/L)	0.71 ± 0.25

**Table 2:** Area Under the Curves (AUC) for proneurotensin and triglycerides after cream and after olive oil

Fat load	No. of participants	Mean AUC		Standard Deviation
**Cream**	22	AUC* Proneurotensin	49	± 74
		AUC** Triglycerides	1.5	± 0.95
**Olive Oil**	22	AUC* Proneurotensin	196	± 115
		AUC** Triglycerides	0.71	± 0.77

Area Under the Curves (AUC) for proneurotensin and triglycerides after Cream and after olive oil

*P < 0.001 for the difference in AUC of proneurotensin between cream and olive oil

**P = 0.004 for the difference in AUC of triglycerides between cream and olive oil

**Fig. 2:**
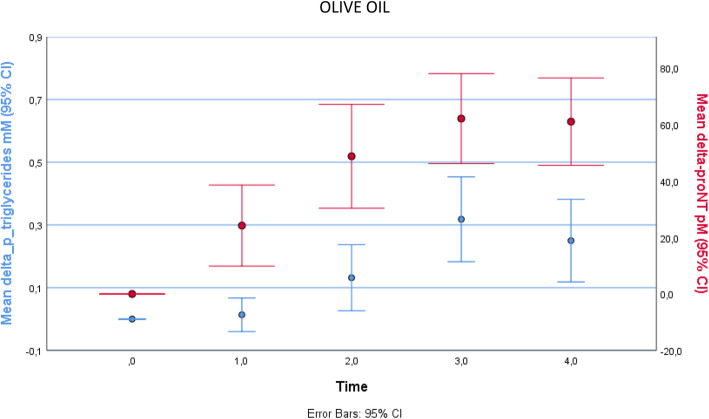
Changes of proneurotensin and triglycerides after ingestion of olive oil and correlation of post lipid load Area Under the Curves (AUC) for proneurotensin with the AUC for plasma triglycerides after olive oil

**Fig. 3:**
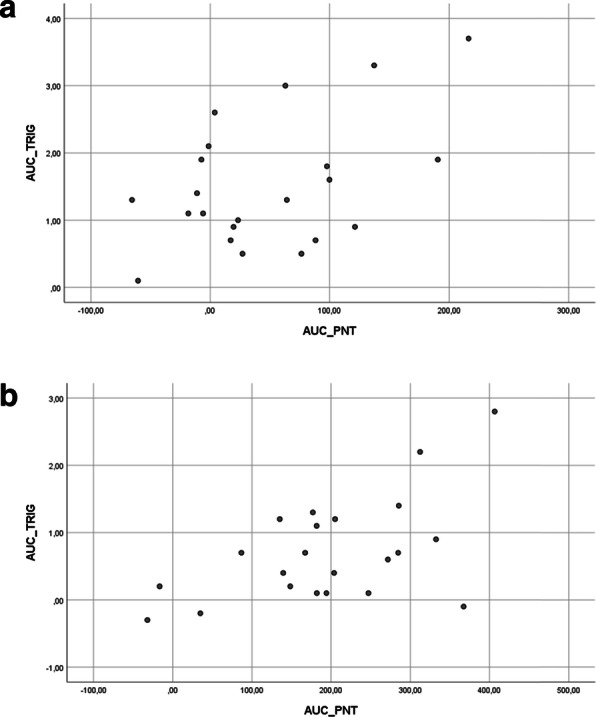
a: Scatter plot graphical presentation of correlation of Area Under the Curves (AUC) for PNT and triglycerides after cream. b: Scatter plot graphical presentation of correlation of Area Under the Curves (AUC) for PNT and triglycerides after olive oil

The post lipid load AUC for proneurotensin correlated significantly with the AUC for plasma triglycerides after both cream (*r* = 0.49*, P = 0.021*) and olive oil (*r* = 0.55*, P = 0.008*) (Fig. 2). The correlation of proneurotensin–AUC and TG-AUC after cream and olive oil has been shown in scattered plot graph in Fig. 3a and b respectively.

In contrast to proneurotensin and triglycerides, plasma glucose concentrations significantly decreased at all-time points after lipid ingestion (after both cream and olive oil) as compared to the fasted plasma glucose levels (Supplementary Table). However, the AUC of glucose was not significantly correlated to the AUC of proneurotensin neither after cream (*r* = − 0.20, *P = 0.36*) nor after olive oil (*r* = − 0.25, *P = 0.25*).

## Discussion

Here it is shown for the first time that there is a relationship between the degree of post-lipid ingestion rise in plasma concentration of proneurotensin and triglycerides in humans, as demonstrated by significant correlation between the post lipid load AUC of proneurotensin and the corresponding AUC of triglycerides after two different forms of lipid loads, i.e. cream and olive oil. This finding extends evidence of a pivotal role of NT in intestinal fat absorption from prior animal studies [9], to suggest a similar role of NT in humans. The cause of the delayed proneurotensin and triglyceride peaks after olive oil vs cream (Fig. 1) is unknown but it is assumed that olive oil, being almost exclusively lipids, results in slower gastric emptying than cream and thus delayed exposure of lipids to intestinal cells secreting proneurotensin.

Previously, high levels of proneurotensin have been shown to predict obesity, diabetes mellitus and CVD, as well as shows relationship with NAFLD [11, 14, 19]. Thus, it is essential to understand mechanisms behind such relationships in order to target the neurotensin system pharmacologically. Although lipid translocation was not measured directly from the intestinal lumen to the blood stream, one can assume that rise in plasma concentration of triglycerides after standardized oral lipid loads more of less exclusively comes from the orally added lipids. Moreover, given the fact that the amount of rise in plasma concentration of proneurotensin after two different oral lipid loads significantly correlated with the rise in plasma triglycerides, it is reasonable to assume that post-lipid ingestion rise of proneurotensin contributes to translocation of the orally added fat from intestinal lumen to the blood stream.

Of note, most of the previous epidemiological studies have examined the associations between fasting level of proneurotensin and cardiometabolic diseases [14, 19, 20], but it is not studied before that proneurotensin release, triggered by fat containing meals have different relationship with the risk of cardiometabolic disease. Studies have shown that high postprandial triglycerides are not only a characteristic feature of diabetes mellitus and insulin resistance but also they are an independent risk factor for future cardiovascular events and might be stronger than the fasting level of triglycerides [21]. Given the link between post-prandial triglyceridemia, CVD risk and current findings of relationship between post-lipid ingestion plasma concentration of proneurotensin and triglycerides, it is proposed that neurotensin contributes to the CVD risk associated with high post-prandial triglycerides. This certainly needs further studies but necessitates discussion on whether proneurotensin levels are modifiable.

The most obvious intervention to reduce postprandial proneurotensin levels is to reduce its trigger, i.e. fat intake. From a pharmacological point of view, the intestinal lipase inhibitor orlistat is of particular interest, which helps in weight reduction by decreasing levels of tumor necrosis factor alpha and interleukin 6 [22] and may reduce the risk of obesity and CVD [23, 24]. Orlistat inhibits both the suggested key effect of neurotensin, i.e. intestinal fat absorption, and intestinal release of neurotensin into the blood stream, the latter being explained by absence of the trigger for neurotensin production and secretion in the enterocyte following orlistat administration, i.e. intracellular fatty acids. It can be proposed in future studies that orlistat therapy can possibly benefit the subjects with high post-prandial proneurotensin and an energy conserving phenotype due to enhanced intestinal fat absorption leading to both reduction in intestinal fat absorption and preventing other potentially harmful effects of high neurotensin like hepatic fat accumulation [9].

Another finding was that glucose significantly decreased after fat ingestion. One explanation for this finding is that neurotensin acts synergistically with glucagon-like peptide-1 (GLP1) and peptide YY (PYY) in the distal small intestines to decrease palatable food intake and to inhibit gastric emptying, thus leading to lower glucose concentration [25]. As a second explanation, it is assumed that glucose production by the liver is reduced after fat ingestion, which could reduce glycemia after lipid ingestion.

### Strengths and limitations

Strengths of the study include the controlled timing and amounts of ingested fat as well as inclusion of healthy study participants with no medications that would potentially bias lipid metabolism. Limitations are that the exact mechanisms by which post-lipid ingestion rise in proneurotensin affects plasma postprandial triglyceride concentrations cannot be explained and no control diets (i.e. protein or carbohydrate) were included, however, the suggested mechanism emanates from the previous animal experimental studies. The study included a rather small number of normal subjects to see the physiological interplay between fat, proneurotensin and triglycerides. There is no prior study to base the assumption of size of the effect estimate on and therefore power calculation was not done. Finally, ELISA (Enzyme-Linked ImmunoSorbent Assay) was not performed to compare the mature hormone (neurotensin) and triglycerides in this study, instead the more stable proneurotensin was used as a surrogate.

## Conclusion

In conclusion, proneurotensin increases after an oral lipid load of both cream and olive oil and the degree of rise of post ingestion triglycerides is significantly related to the rise of post ingestion plasma proneurotensin. Findings of this study can be of importance for future treatment strategies for metabolic diseases and CVD through neurotensin related mechanisms.

## Supplementary information

**Additional file 1: Table S1.** Changes in plasma glucose concentration at all-time points both after cream and after olive oil ingestion.

## Data Availability

Data analysis are included in the published article and is available on request.

## Abbreviations

NT: Neurotensin
T2D: Type 2 diabetes
CVD: Cardiovascular Disease
BMI: Body mass Index
SD: Standard deviations
AUC: Area under the curve
TG: Triglycerides
ELISA: Enzyme-Linked ImmunoSorbent Assay
