# Supplementary material for: Magnitude of rise in proneurotensin is related to amount of triglyceride appearance in blood after standardized oral intake of both saturated and unsaturated fat
Source: Lipids Health Dis. 2020 Aug 21;19:191. doi: 10.1186/s12944-020-01361-0 (PMC7441720; doi:10.1186/s12944-020-01361-0)
Supplement: Supplementary file 1 — Additional file 1: Table S1. Changes in plasma glucose concentration at all-time points both after cream and after olive oil ingestion. [file 12944_2020_1361_MOESM1_ESM.docx]

| **Δ Glucose** | **Cream** | | | **Olive Oil** | | |
| --- | --- | --- | --- | --- | --- | --- |
| **Time (h)** | **Mean** | **95% (CI)**  **Confidence Interval** | **P-value** | **Mean** | **95% (CI)**  **Confidence Interval** | **P-value** |
| **1 h** | - 0.54 | ( - 0.75 - - 0.32) | 0.000 | - 0.39 | ( - 0.58 - - 0.20) | 0.000 |
| **2 h** | - 0.33 | ( - 0.54 - - 0.11) | 0.005 | - 0.42 | ( - 0.64 - - 0.20) | 0.001 |
| **3 h** | - 0.30 | ( - 0.46 - - 0.13) | 0.002 | - 0.31 | ( - 0.51 - - 0.11) | 0.004 |
| **4 h** | - 0.28 | ( - 0.49 - - 0.07) | 0.011 | - 0.29 | (- 0.46 - - 0.11) | 0.003 |

**Supplementary Table: Changes in plasma glucose concentration at all-time points both after cream and after olive oil ingestion.**
